# An Unexpected Cause of Chest Pain While Self-Pleasuring: A Ripping Doom Excitement

**DOI:** 10.7759/cureus.38436

**Published:** 2023-05-02

**Authors:** Jose Escabi-Mendoza, Porfirio E Diaz-Rodriguez, Diego H Gonzalez-Bravo, Eduardo Partida-Rodriguez

**Affiliations:** 1 Cardiovascular Disease, Veterans Affairs (VA) Caribbean Healthcare System, San Juan, PRI

**Keywords:** acute cardiac care, aorta, aortic dissection, cardiac troponin, ecg, cardiac chest pain, type a aortic dissection

## Abstract

Acute aortic dissection (AD) involves the tearing of the aortic intima by shearing forces, resulting in a false lumen, which, depending on its location and extent, may lead to hemodynamic compromise, hypoperfusion of vital organs, or even rupture of the aorta. The classical presentation is a sudden chest or back pain described as sharp or ripping in quality. We present a 60-year-old male with a history of hypertension, Liddle’s syndrome, obstructive sleep apnea, and chronic cannabis use for insomnia who arrived at a non-PCI hospital complaining of severe retrosternal chest pain lasting several hours in evolution that started upon masturbation. The pain was ripping in character, starting retrosternally and radiating to his neck and back. After evidence of rising troponin values, he was initially diagnosed with non-ST segment elevation myocardial infarction (NSTEMI), managed with dual antiplatelet therapy with full anticoagulation, and subsequently transferred to our institution for further care. Shortly after his arrival at our hospital, he suddenly deteriorated with recurrent chest pain and hypotension, which triggered an emergent bedside echocardiogram evaluation. This revealed a hemodynamically significant pericardial effusion, moderate to severe aortic valve regurgitation (AR), and an intimal flap visualized on the ascending and descending aorta, suggestive of an extensive AD. A computerized tomographic angiogram confirmed the diagnosis of a Stanford type A AD that required an emergent surgical pericardiotomy, ascending aorta with partial arch replacement, and aortic valve repair. Often, AD may mimic an acute coronary syndrome (ACS) or even present with an acute myocardial infarction (AMI). The appropriate diagnostic imaging evaluation prior to the initiation of anticoagulation therapy should be done in patients with higher-risk clinical criteria for AD to reduce adverse treatment outcomes. The use of a simple three-step diagnostic algorithm for acute aortic syndromes (AAS) may decrease diagnostic delays, misdiagnosis, and inappropriate therapies.

## Introduction

Acute aortic syndrome (AAS) describes a range of severe, life-threatening abnormalities of the aorta that occur within less than two weeks of the initial clinical presentation. It includes classic aortic dissection (AD; the most common type), intramural aortic hematoma, and penetrating aortic ulcer. They all have a similar clinical presentation, prognosis, and management according to their location (involvement of the ascending aorta) or the presence of complications. Acute AD involves tearing between the aortic intimomedial wall layers by shearing forces of the aortic pressure pulsation, resulting in the creation and propagation of a false lumen within the medial layer (as the favored hypothesis) [1-2]. Depending on the location and extent of the AD, it may lead to hemodynamic compromise, malperfusion of vital organs, or other serious complications (cardiac tamponade, severe aortic regurgitation [AR], or even rupture of the aorta). The classical presentation of AD is a sudden chest or back pain described as sharp or ripping in quality. Up to 20% of the patients die before arriving at the hospital, and every hour without surgical treatment, mortality increases by 1-2%; therefore, early recognition and treatment are of crucial importance [1]. According to the largest national database in the United States, while contemporary data suggest an increase in AAS recognition, in-hospital mortality (26%) has not changed over time [2], possibly related to the low availability of specialized aorta centers to better treat this complex, life-threatening condition. This case was presented at the Puerto Rico Congress of Cardiology as a poster presentation on October 15-18, 2020.

## Case presentation

**Figure 1:**
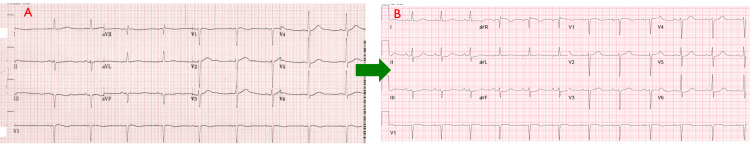
ECG upon arrival and after developing hypotension and chest pain (A) Initial ECG upon arrival with normal sinus rhythm and no ST-segment changes suggestive of acute ischemia. (B) ECG related to recurrent chest pain and marked hypotension, showing non-specific ST changes in leads V5-V6.

A 60-year-old male with a history of hypertension, Liddle’s syndrome, obstructive sleep apnea, and chronic cannabis use for insomnia. Home medications for his resistant hypertension included amiloride, extended-release diltiazem, lisinopril, doxazosin, and metoprolol, with questionable compliance. He arrived at another institution with a complaint of severe retrosternal chest pain lasting several hours. The pain started upon masturbation and within an hour of smoking cannabis. It was described as ripping in character, retrosternal, and radiating to both sides of his neck and back. Initially managed with aspirin, ticagrelor, and enoxaparin for a presumed diagnosis of a non-ST segment elevation myocardial infarction (NSTEMI) and subsequently transferred to our institution. Upon arrival in our emergency department (ED), he had no chest pain, was afebrile, and was hemodynamically stable, with a blood pressure of 174/68 mmHg, a heart rate of 59 beats per minute, a respiratory rate of 18 breaths/min, height of 71 inches, and a weight of 223 pounds. The physical exam was consistent with warm extremities, no focal neurological deficits, symmetrically strong peripheral arterial pulses, a mild systolic ejection murmur at the lower left sternal border (LLSB), no crackles, and no jugular venous distension, yet bilateral carotid bruits were present. Serial high-sensitive troponins were abnormal and with an increasing delta trend: 49, 56, 69, and 136 ng/L at 0/1/3/6 hours, respectively (abnormal cut-off >22 ng/L). His initial 12-lead electrocardiogram (ECG) upon arrival presented with a normal sinus rhythm and without acute ST-segment or T-wave ischemic changes (Figure 1).

**Figure 2:**
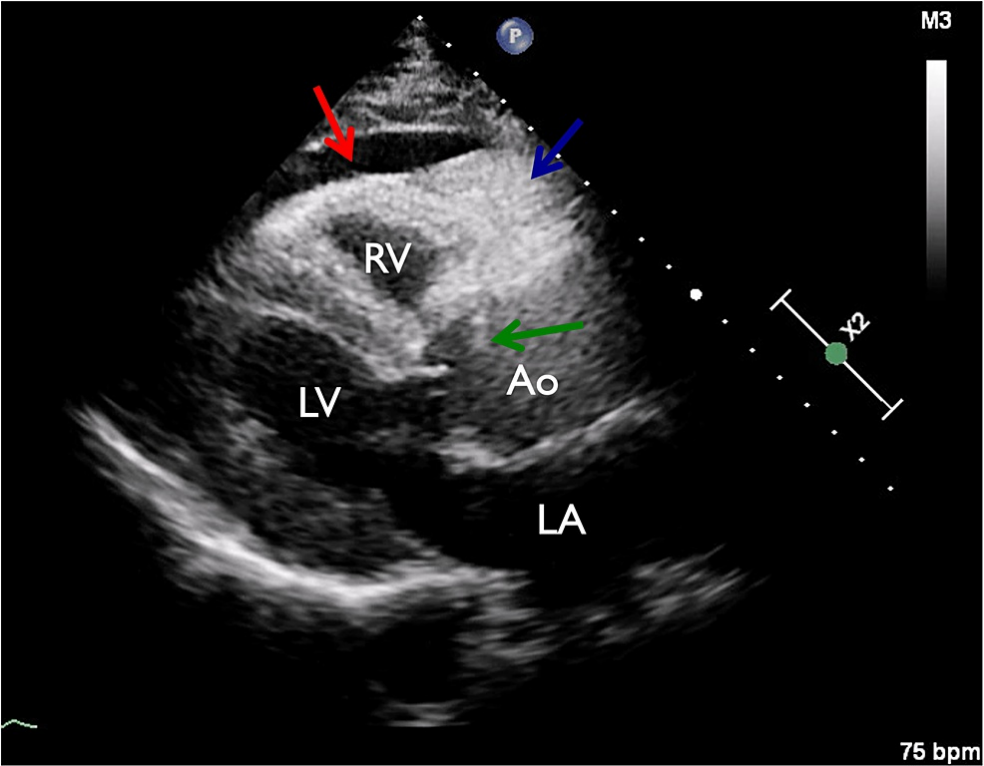
Bedside parasternal long axis view (PLAX) Bedside PLAX showing dilated ascending aorta with intimal flap (green arrow). There is a moderate pericardial effusion (red arrow) with associated pericardial coagulum (blue arrow). RV: right ventricle, Ao: aorta, LV: left ventricle, LA; left atrium.

**Figure 3:**
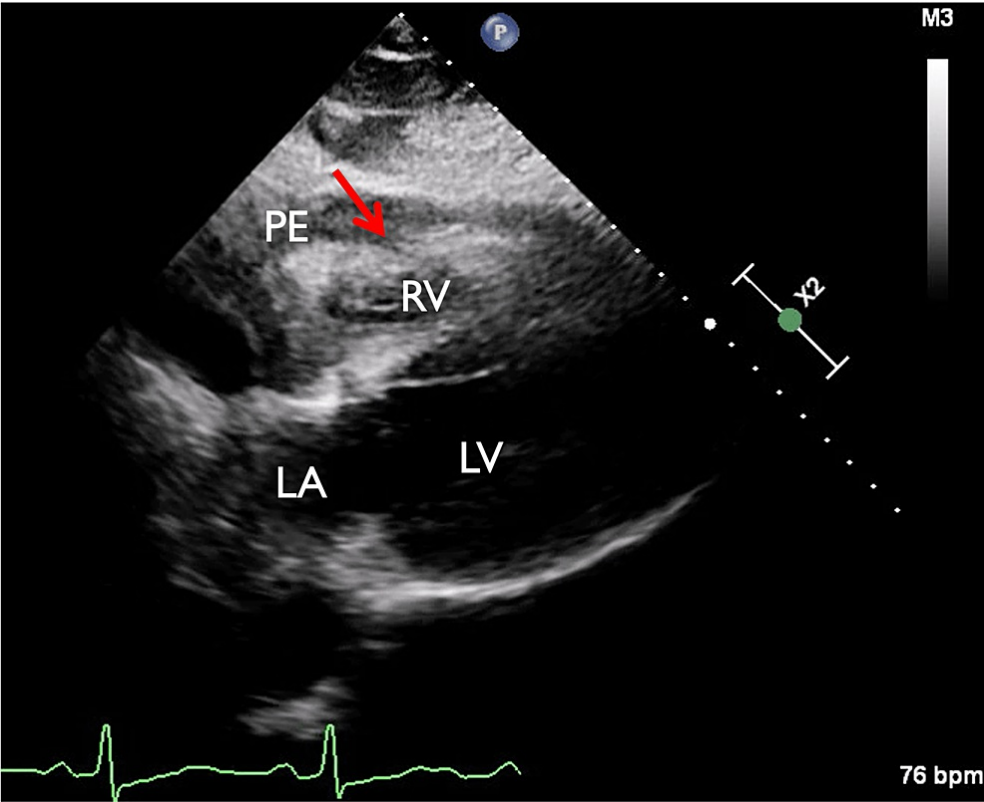
Bedside subcostal view disclosing right ventricular diastolic collapse Red arrow showing right ventricular diastolic collapse. PE: pericardial effusion, RV: right ventricle, LV: left ventricle, LA: left atrium.

**Figure 4:**
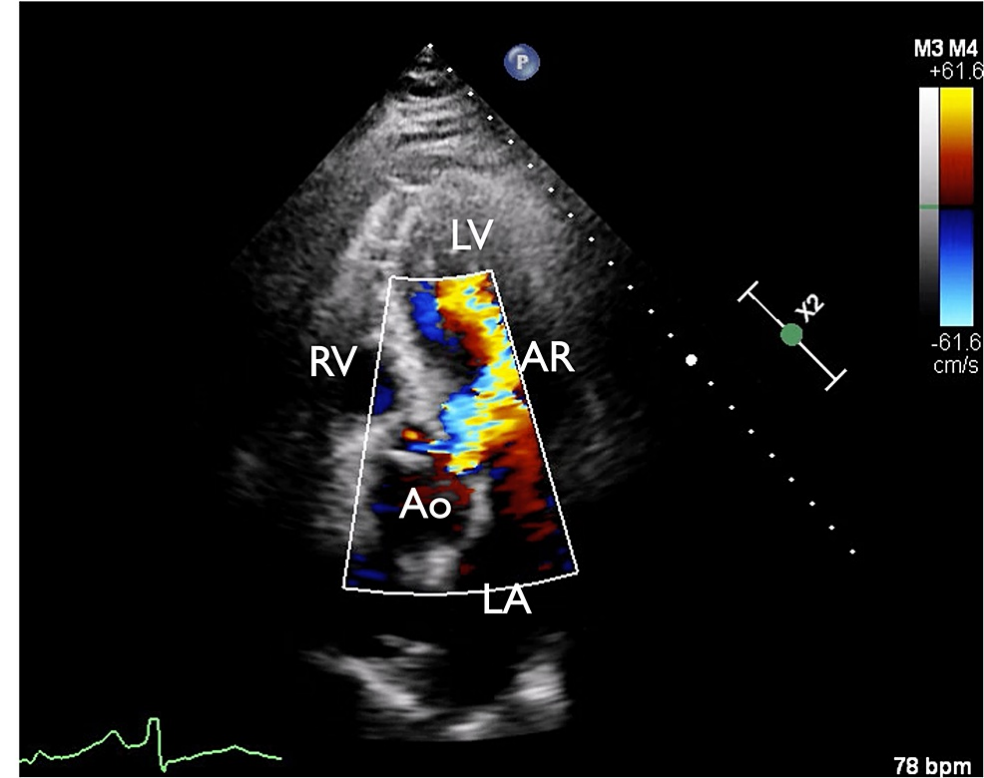
Apical 5-chamber view Apical 5-chamber view with color where one can appreciate a moderate-to-severe aortic valve insufficiency jet extending into the left ventricular apex. It is most likely secondary to dissection flap prolapsing into the valve and causing poor coaptation. Ao: aorta, LV: left ventricle, AR: aortic regurgitation, RV: right ventricle, LA: left atrium.

**Figure 5:**
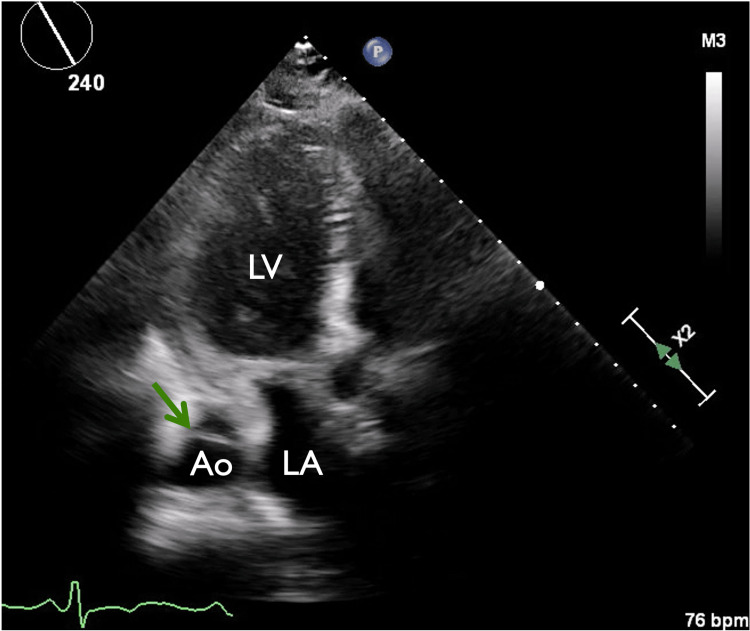
Apical 3-chamber view revealing descending thoracic aorta with intimal flap Descending thoracic aorta with intimal flap (green arrow). LV: left ventricle, Ao: aorta, LA: left atrium.

**Figure 6:**
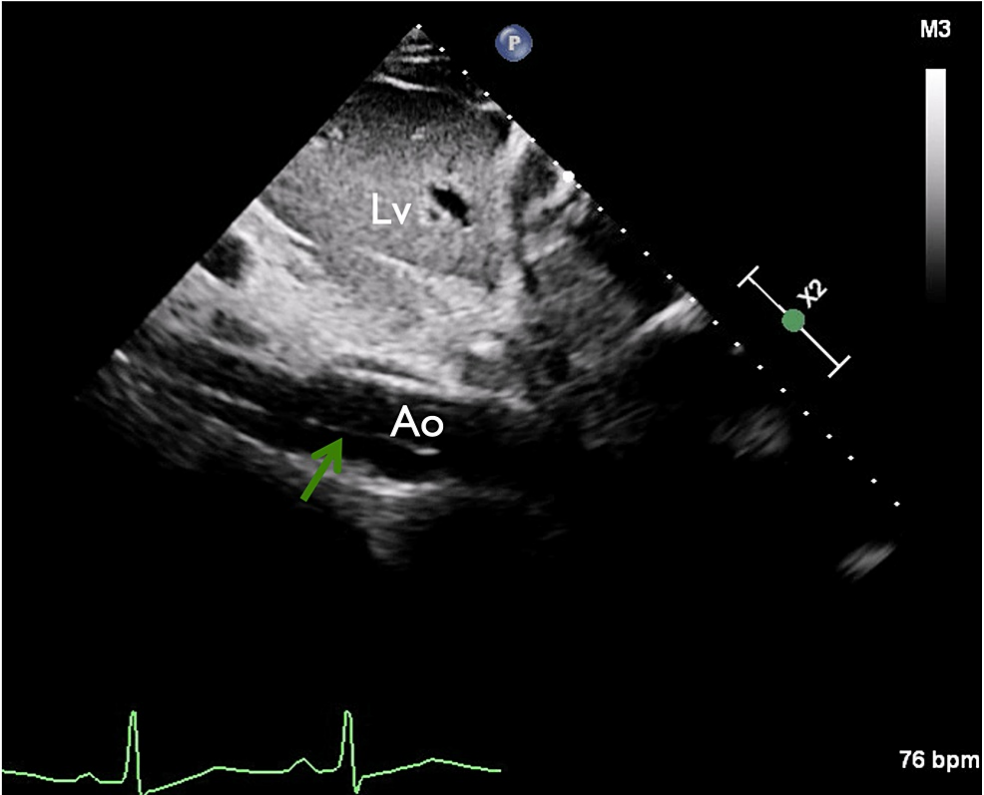
Subcostal view of the abdominal aorta showing the intimal flap Abdominal aorta intimal flap (green arrow). Lv: liver, Ao: abdominal aorta.

**Figure 7:**
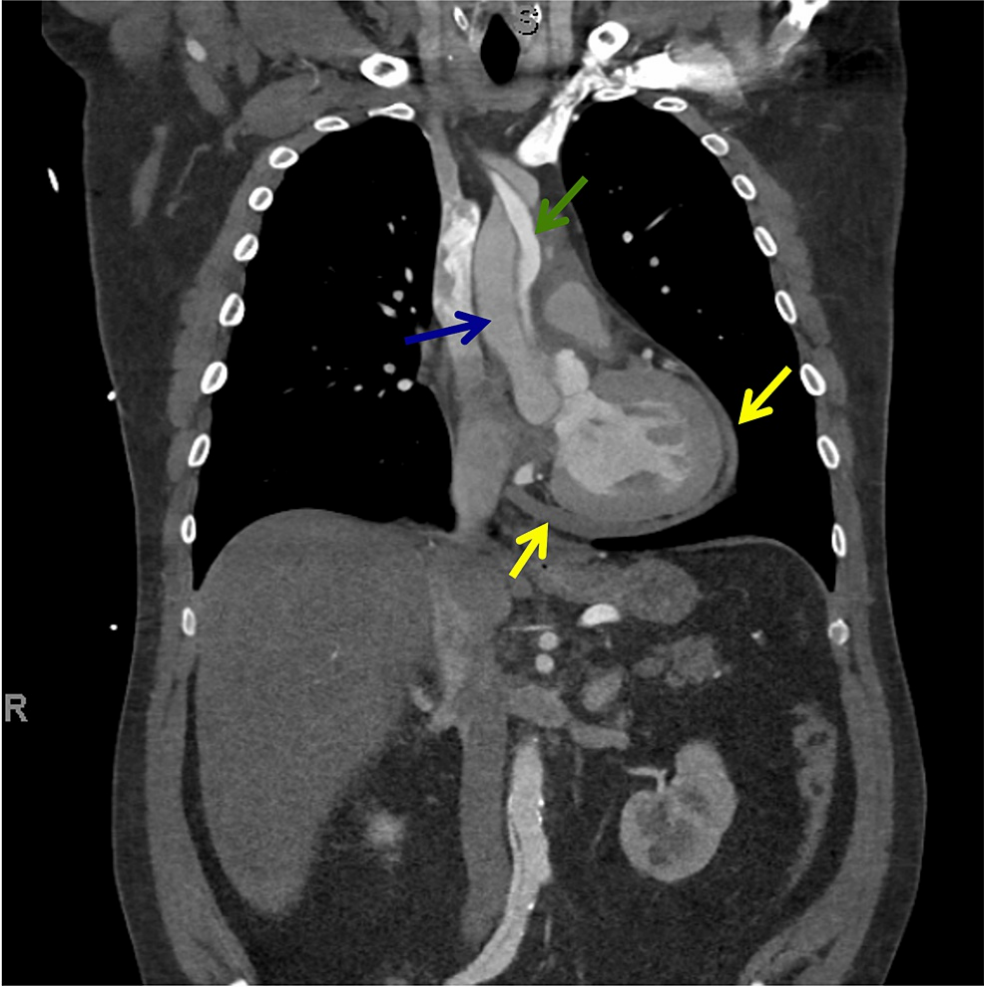
Coronal section of CT angiogram showing extensive ascending thoracic aorta dissection (Stanford type-A AD) which extended to the right Iliac artery and a moderate pericardial effusion The more darker lumen represents the false lumen (blue arrow). The brighter lumen represents the true lumen (green arrow). Pericardial effusion is evident (yellow arrows).

**Figure 8:**
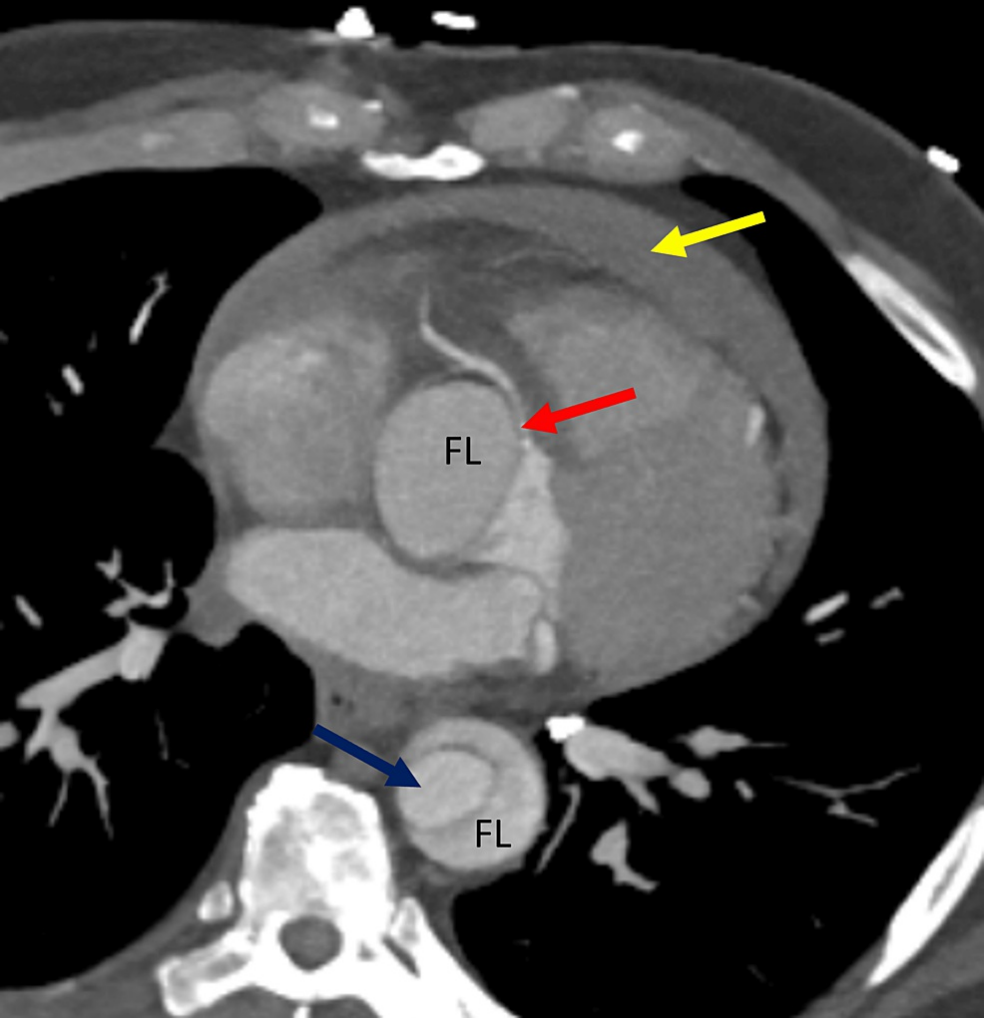
Axial section showing ascending thoracic aorta dissection sparing the right coronary ostium and extending into the descending thoracic aorta Aortic dissection extending into the descending thoracic aorta (blue arrow). Right coronary artery ostium in the true lumen, spared by the dissection (red arrow). A moderate pericardial effusion is also evident anteriorly (yellow arrow). FL: false lumen.

**Figure 9:**
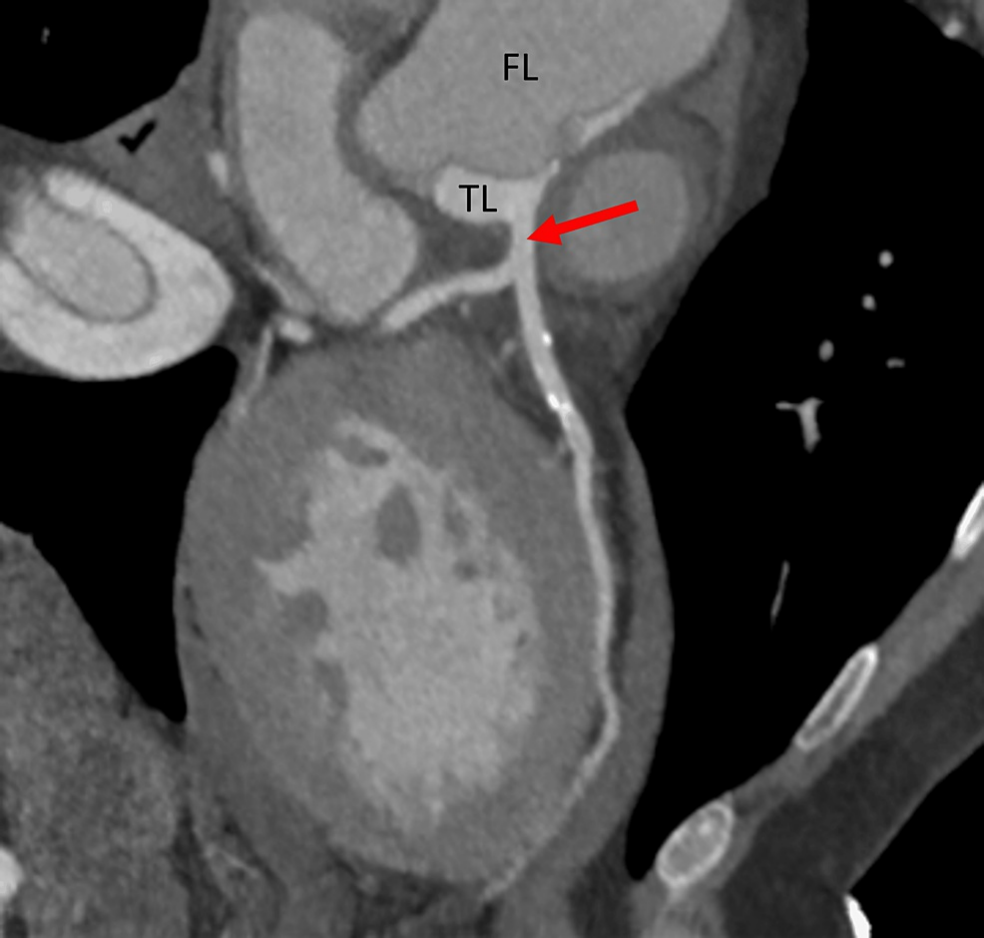
CT showing ascending aorta dissection sparing the left coronary ostium Left main coronary spared from the aortic dissection (red arrow). FL: false lumen, TL: true lumen.

**Figure 10:**
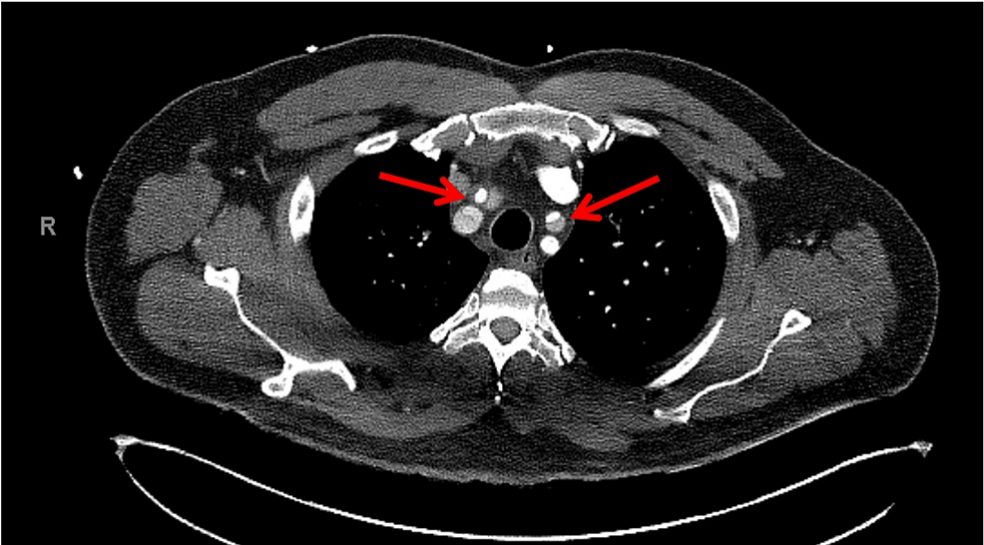
Axial section dissection extending into the right and left common carotid arteries Dissection shown extending into the right and left common carotid arteries (red arrows).

In view of chest pain associated with positive cardiac biomarkers, he was admitted to the Coronary Care Unit on intravenous nitroglycerin. While waiting for his admission, he suddenly had recurrent chest pain associated with marked hypotension (75/43 mmHg), for which the intravenous nitroglycerin was discontinued, fluid resuscitation was provided, and he was temporarily started on intravenous norepinephrine. The on-call cardiology team was contacted for an emergent evaluation. Subsequent ECG showed nonspecific ST-segment changes in leads V5-V6 (Figure 1). An emergent bedside echocardiogram revealed a moderate pericardial effusion of hemodynamic significance (Figure 2), as suggested by partial right ventricular chamber collapse (Figure 3) early in diastole and a plethoric non-collapsing inferior vena cava. The pericardial effusion also contained echogenic strands, which are highly concerning for blood clots. Additionally, a moderate to severe aortic valve insufficiency (Figure 4) was present according to color Doppler findings and pressure half-time assessment (315 ms). An intimal flap was visualized on the ascending and descending aorta, suggesting an extensive AD (Figures 2-6). A computer tomographic angiogram (CTA) of the chest and abdomen revealed a large pericardial effusion with an extensive AD extending from the level of the ascending aorta into his right brachiocephalic artery, the right and left common carotid arteries, and inferiorly to the right iliac artery (Figures 7-10), although not occlusive in diameter (or clinically). These findings were consistent with an extensive Stanford type-A AD with rupture into the pericardium, for which volume expansion and vasopressors were provided and emergently operated on the same day. The surgery included an open pericardiotomy with pericardial blood drainage, replacement of the ascending aorta from just above the commissure to the take-off of the innominate artery, partial replacement of the aortic arch, and aortic valve repair with successful results. Multiple intraoperative packed red blood cell units (8), fresh frozen plasma units (4), cryoprecipitate units (10), and platelet apheresis (3) were provided in view of bleeding complications related to the arrival provision of dual antiplatelet and anticoagulation therapy prior to the diagnosis of AD.

## Discussion

AAS such as AD, intramural aortic hematoma, and penetrating aortic ulcer are life-threatening cardiovascular emergencies affecting 4-7/100,000 person-years in more contemporary large population-based cohort studies [1]. Misdiagnosis rates are high, reaching up to 39% because of unspecific clinical presentations [1]. This can have a detrimental impact on patients' outcomes when surgical treatment is delayed. Patients with AAS may be classified into two categories depending on the involvement (Stanford type-A) or absence (Stanford type-B) of the ascending aorta from a surgical and prognostic standpoint. In general, patients with type-A AAS are considered for emergency open surgical repair. Surgical urgency also depends on the presence of ongoing complications related to malperfusion of vital organs or, as in this case with the presence of hemodynamic compromise from cardiac tamponade and acute AR. The goals of surgery are to correct hemodynamic instability, correct and prevent organ malperfusion, and prevent aortic rupture and death. In most cases, this can be achieved by ascending aorta and hemiarch replacement and aortic valve resuspension. As usual, the benefits of surgery must be weighed against the risks of the surgery itself, using sound clinical judgment and the patient’s expectations. Initial management, consisting of aggressive blood pressure (SBP <120 mmHg) and heart rate control (HR = 60-80), should be provided prior to surgery to decrease aortic wall stress and avoid the progression of the dissection. Sedatives and analgesia should also be considered.

**Figure 11:**
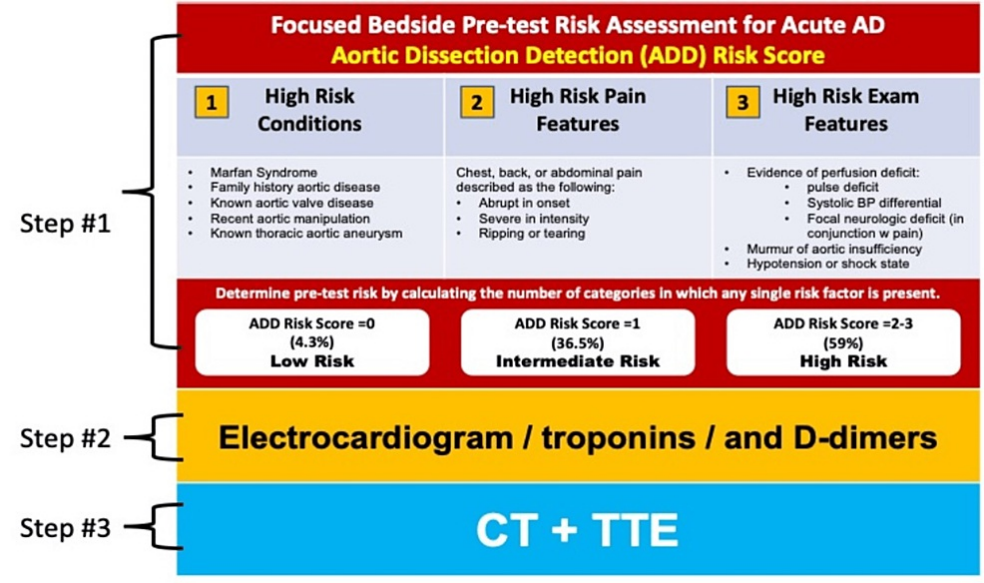
Suggested three-step diagnostic algorithm for patients with a suspicion of AAS Our modified diagnostic algorithm for patients with suspicion of AAS. ADD: aortic dissection detection, CT: computed tomography, TTE: transthoracic echocardiography [2,7].

The aortic dissection in this patient could have been triggered by a myriad of factors. First, he had Liddle’s syndrome, which exposes the aorta to chronic hypertension, one of the classical risk factors for dissection. This syndrome is a rare genetic disorder inherited in an autosomal dominant manner and is characterized by early and frequently severe arterial hypertension. This results in a hyperaldosteronism-like state (pseudo-aldosteronism), but with a low renin-low aldosterone state. In these patients, target organ dysfunction is not an uncommon complication in view of the early onset and longstanding resistance to hypertension. While the mean age of male patients with classic AD ranges from 66 to 72 years, the presence of Liddle’s syndrome associated with uncontrolled hypertension would explain why this patient presented with AD close to a decade sooner than the average. Abbass et al. described a patient with Liddle’s syndrome presenting with an AAS of the thoracic descending aorta [3]. Second, sexual activity has been linked to aortic dissection, possibly due to a rise in catecholamine levels causing a significant elevation of blood pressure and thus sheer stress to the aorta [4]. One retrospective study by Gansera et al. showed that the physical and emotional stress associated with sexual activity was a meaningful promoter of aortic dissection in 11% (p=0.03) of younger males (<60 y/o) presenting with this diagnosis [5]. Third, even though marijuana is the most commonly used illicit drug in the US, its relationship to AAS has not been well studied. Marijuana has been shown to increase heart rate, blood pressure, and systemic sympathetic tone. Sarmiento et al. performed a retrospective review of 152 consecutive patients with acute Stanford type-A AD, of whom 51 (34%) underwent comprehensive urine toxicology screening upon clinical presentation. Of these, 18% returned positive results for tetrahydrocannabinol (THC), a positivity rate markedly higher than would be expected in the general population. The THC patients were significantly younger than the non-THC patients (mean age of 48 versus 61 years, respectively, P=0.004). These results suggest that marijuana may be a contributing risk factor for acute type-A AD, although its pathophysiological mechanisms leading to dissection remain elusive [6-7]. Most likely, the AD in our current case resulted from the interplay of his chronic resistant hypertension secondary to Liddle’s syndrome, leading to atherosclerosis and aortopathy, combined with strong catecholamine triggers (masturbation after smoking marijuana), contributing to increased sheer stress to the aorta and creating the perfect scenario for the development and propagation of AD. For accurate diagnosis of AAS, Vilacosta et al. in a state-of-the-art review recommended the use of a three-step diagnostic algorithm [2]. Step 1 is devoted to calculating the pretest probability of AAS based on the aortic dissection detection risk score (ADD-RS), which consists of 12 risk markers classified into three categories: predisposing conditions, pain features, and physical exam findings (Figure 11) [2,8]. For each category, 1 point can be awarded if at least one risk marker in the category applies. A score of 0 indicates low risk for AAS; if the score is 1, the risk is intermediate; and if it is 2-3, the risk of having an AAS is high. Step 2 is a key stage, with the performance of an ECG, chest X-ray, and laboratory testing including troponins and D-dimers. So, in patients with "aortic pain," the triad of normal ECG, normal troponins, and increased levels of D-dimers is a warning pattern of AAS. Plasma D-dimers (DD) have a high sensitivity for diagnosing AAS and correlate significantly with the extension of AD. However, its most significant value is its high negative predictive value, where you can consistently rule out the diagnosis of AAS with normal results (<500 ng/mL). Troponin elevation does not absolutely rule out AAS (as discussed below). In step 3, the diagnosis of AAS is confirmed or excluded by performing a CTA scan of the entire aorta. Focused transthoracic echocardiography (TTE) in the emergency room can be useful for the diagnosis as well as for assessing serious complications from AD with involvement of the ascending aorta, as in the current case that presented with a severe AR and cardiac tamponade.

In one multicenter and prospective observational study done by Nazerian et al., patients with suspected AAS with a low or intermediate risk (ADD-RS = 0-1) plus a normal DD had a sensitivity (for having AAS) and a negative predictive value (for not having AAS) found to be very high in 98.8% and 99.7%, respectively, thus helpful for effectively ruling out such a diagnosis [9] without the need for CTA evaluation. Determination of a low pre-test probability will improve resource utilization (by avoiding unnecessary imaging studies), while an intermediate to high pre-test probability will lead you to pursue further diagnostic imaging evaluation for the diagnosis of AD. Alternatively, in cases deemed high-risk (ADD-RS = 2-3), one should proceed directly to confirmatory testing with a CTA or transesophageal echocardiogram irrespective of DD levels, in view of the high sensitivity and >95% likelihood of having AD. Our patient was at high risk for AAS (ADD-RS=2), for which a CTA evaluation irrespective of DD levels was recommended and performed immediately to confirm the diagnosis. AAS can have the same presentation as acute coronary syndrome (ACS). Up to 40% of patients with AAS can have non-specific ST-segment changes, raising the possibility of ischemia and ACS/NSTEMI [10]. Acute myocardial infarction (AMI) with ST-segment elevation related to the extension of the dissection flap into the ostium of the coronary artery in acute type-A AD develops in 1-2% of cases. Troponin elevation in AD is not infrequent and mostly related to a non-atherothrombotic coronary process, such as dissection extension into either the coronary ostium leading to coronary hypoperfusion or occlusion and/or myocardial oxygen demand/supply mismatch related to hemodynamic alterations from AD complications. In patients with AAS misdiagnosed as ACS, the employment of anticoagulation and antiplatelet agents can theoretically have lethal consequences [11] by worsening and fueling the dissection process. Therefore, making the distinction early in the natural history of the disease is of uttermost importance, as in the case of our patient. Transthoracic echocardiography and point-of-care ultrasound (POCUS) are powerful tools that will certainly aid the clinician in making the distinction between AAS and ACS. In a retrospective study by Pare et al., the use of POCUS was associated with a faster diagnosis of AAS (median of 80 vs. 226 minutes, p = 0.023), a lower rate of misdiagnosis (0% vs. 43.8%, p = 0.028), and lower mortality (15.4% vs. 37.5%, p=0.27) [12]. A survey in the United Kingdom gathered 175 ED consultants across 70 hospital trusts in which they simulated clinical scenarios that mimicked ACS in the early stages, where AD could simultaneously be the diagnosis [13]. Survey responses revealed that in a patient with acute type-A AD presenting with chest pain and elevated cardiac markers, there is a high probability of ACS treatment being commenced and a significant risk of failing to request the necessary imaging to diagnose AD. The lack of use of an AD algorithm was the strongest predictor of clinicians avoiding the use of more definitive investigations for acute type-A AD (OR=0.31).

Our case emphasizes the importance of early employment of echocardiography in making the distinction between AAS and ACS. An early distinction can guide the clinician in determining whether providing antithrombotic agents is safe and will not hasten a possible AAS.

## Conclusions

Acute AD is potentially a lethal medical emergency and may mimic or present with a concomitant AMI. A high level of clinical suspicion with the use of a three-step diagnostic algorithm for AAS and early POCUS in the evaluation of these patients can expedite the diagnosis and prevent unnecessary antithrombotic therapies that can adversely affect AD extension and patient outcomes.

## References

[1] Isselbacher EM, Preventza O, Hamilton Black J 3rd (2022). 2022 ACC/AHA guideline for the diagnosis and management of aortic disease: a report of the American Heart Association/American College of Cardiology joint committee on clinical practice guidelines. Circulation.

[2] Vilacosta I, San Román JA, di Bartolomeo R (2021). Acute aortic syndrome revisited: JACC state-of-the-art review. J Am Coll Cardiol.

[3] Abbass A, D'Souza J, Khalid S, Asad-Ur-Rahman F, Limback J, Burt J, Shah R (2017). Liddle syndrome in association with aortic dissection. Cureus.

[4] Ridha A Ridha A,  Safiullah S  Safiullah S, Al-Abayechi S Al-Abayechi S,  Ghadai A  Ghadai A, Nadeem AU Nadeem AU (2015). A rare presentation of a life- threatening condition secondary to masturbatory activity: a case report. J Cardiovasc Disord.

[5] Gansera L, Deutsch O, Szameitat L, Eichinger W, Gansera B (2016). Aortic dissections type A during sexual intercourse in male patients: accident or systematic coincidence? Examination of 365 patients with acute aortic dissection within 20 years. Thorac Cardiovasc Surg.

[6] Mason EK, Gak AE, Finno JG, Cannon RD, Jacoby JL (2019). Thoracic aortic dissection associated with marijuana use. J Emerg Med.

[7] Sarmiento IC, Giammarino A, Scheinerman SJ, Guirola A, Hartman A, Brinster D, Hemli JM (2021). Marijuana: An underappreciated risk factor for acute type A aortic dissection?. Heart Surg Forum.

[8] Rogers AM, Hermann LK, Booher AM (2011). Sensitivity of the aortic dissection detection risk score, a novel guideline-based tool for identification of acute aortic dissection at initial presentation: results from the international registry of acute aortic dissection. Circulation.

[9] Nazerian P, Mueller C, Soeiro AM (2018). Diagnostic accuracy of the aortic dissection detection risk score plus D-dimer for acute aortic syndromes: the advised prospective multicenter study. Circulation.

[10] Tolefac PN, Dzudie A, Mouliom S (2018). Acute type A aortic dissection involving the iliac and left renal arteries, misdiagnosed as myocardial infarction. Cardiovasc J Afr.

[11] Zschaler S, Schmidt G, Kukucka M, Syrmas G, Zaschke L, Kurz SD (2018). How to prevent inadvertent emergency anticoagulation in acute type A aortic dissection: when in doubt, don't. Cardiovasc Diagn Ther.

[12] Pare JR, Liu R, Moore CL, Sherban T, Kelleher MS Jr, Thomas S, Taylor RA (2016). Emergency physician focused cardiac ultrasound improves diagnosis of ascending aortic dissection. Am J Emerg Med.

[13] Salmasi MY, Hartley P, Hussein M, Jarral O, Pepper J, Nienaber C, Athanasiou T (2020). Diagnosis and management of acute Type-A aortic dissection in emergency departments: results of a UK national survey. Int J Cardiol.

